# Impacts of financial development and green trade on the shadow economy: An insight of eagle countries using Bayesian approaches

**DOI:** 10.1371/journal.pone.0303135

**Published:** 2024-05-28

**Authors:** Bui Dan Thanh, Nguyen Van Diep, Nguyen Huynh Mai Tram

**Affiliations:** 1 Faculty of Postgraduate Education, Ho Chi Minh University of Banking, Ho Chi Minh City, Vietnam; 2 Faculty of Finance and Banking, Ho Chi Minh City Open University, Ho Chi Minh City, Vietnam; 3 Ho Chi Minh City Open University, Ho Chi Minh City, Vietnam; Cavendish University / Kyambogo University, UGANDA

## Abstract

The existence of a shadow economy is recognized as an impediment to sustainable development. By applying the Bayesian approaches, the current article investigates the linkage between financial development, green trade, and the scope of the shadow economy, aiming to contribute to a comprehensive understanding of how these factors address the challenge posed by the shadow economy in Emerging and Growth-Leading Economies (EAGLE) from 2003 to 2016. The results demonstrate that *(i)* The progress of the financial sector is expected to diminish the scale of the shadow economy. Specifically, the expansion of financial institutions and markets has a strong and negative influence on the shadow economy. *(ii)* Increased involvement in green trade is likely to result in a decreased shadow economy. Empirical findings provide evidence for effective policymaking in simultaneously promoting sustainable trade practices, strengthening financial systems, and curtailing informal economic activities for inclusive economic development.

## 1. Introduction

The global phenomenon known as the shadow economy also referred to as the gray, informal, cash, hidden, underground, or black economy, has been a persistent concern over an extended period [1–3]. Encompassing concealed activities that escape public authorities’ scrutiny because of regularity, monetary transactions, or institutional reasons, the shadow economy diminishes the tax base, leading to a reduction in the country’s tax revenue and subsequently exerting adverse effects on economic growth [4, 5]. These covert transactions, not factored into the official Gross Domestic Product (GDP), result in a misrepresentation of macroeconomic indicators crucial for the country’s economic policymaking [6–8]. Researchers have investigated factors affecting the activities of the shadow economy, for example, foreign direct investment [9, 10]; institutional quality [11–14]; and globalization [15–17].

Among various factors, previous studies have indicated that financial development is a significant institutional determinant influencing the activities of shadow economics, primarily caused by financial accessibility [18, 19]. The formal sector receives financial support from the financial system through the transfer of funds from lenders to borrowers, and it also provides various financial services, encouraging businesses to transition into the official areas [20, 21]. The expansion of the financial sector diminishes occurrences of evading taxation, oversees business activities to facilitate tax compliance, and lowers the prevalence of activities in the shadow economy (Khan & Rehman, 2022). The financial sector serves various economic roles, notably providing businesses with essential funds, minimizing financial obstacles, and overseeing business transactions related to taxation. Additionally, the integration of technology, such as digitalization in finance or the use of financial technology, plays a crucial role in the development of the financial sector, particularly in banking [22, 23]. This technological advancement can have implications for the prevalence of shadow economic activities and influence overall sectoral growth [24].

Simultaneously, the rising tide of “green trade” reflects a global shift towards environmentally sustainable practices. In the contemporary era, nations are increasingly fostering the growth of an environmentally friendly economy [25, 26]. Rather than engaging in the conventional trade of a diverse range of products and services, there is a heightened focus on green trade, which involves the trade of products and services that meet specific environmental standards and criteria. In essence, green products can be categorized into two main groups: “traditional environmental goods” and “environmentally preferable goods” [27, 28]. The first type, traditional environmental goods, includes products designed to address environmental issues, such as equipment for controlling air pollution. The second type, environmentally preferable products, refers to goods that have a lower environmental impact than their counterparts either during manufacturing or throughout their life cycle, for example, solar cars and jute bags [29]. The advantages of the latter kind of product become evident during various stages of their life cycle, including production, use, and disposal [30]. One potential influence of green trade on the scope of the shadow economy is the creation of new opportunities and markets. The shift towards eco-friendly practices may stimulate innovation and entrepreneurship, leading to the emergence of legitimate businesses operating within the framework of green trade [31, 32]. This, in turn, could contribute to a diminution in the magnitude of the shadow economy as more economic activities transition towards the formal sector. Conversely, the adoption of green trade practices might pose challenges to certain sectors within the shadow economy. Industries engaged in environmentally detrimental activities may face increased scrutiny and regulation, potentially limiting their operations and pushing some into the informal realm to evade such constraints [33, 34]. This could result in a transformation of the shadow economy rather than its outright reduction. Moreover, the integration of green trade practices could impact employment patterns within the shadow economy [35, 36]. As legitimate green businesses grow, there may be opportunities for workers in informal sectors to transition to more formal and sustainable employment. However, for those heavily reliant on environmentally harmful activities, the transition may exacerbate existing challenges.

Emerging and Growth-Leading Economies (EAGLES) are estimated to contribute more to global economic growth than the average contribution made by Western nations (notably the United States and the European Union) [37]. Their primary impact on the world economy stems from strong growth in domestic demand, an impressive capacity to attract foreign direct investment, and the increasing technological prowess resulting from a more educated human capital. Additionally, their expanding businesses are demonstrating competitiveness and innovation on the global stage, further enhancing their global economic influence. Emerging markets are projected to account for 73% of worldwide growth from 2013 to 2023, with EAGLE making a significant contribution of 51%, the members of Nest group contributing 14%, and other emerging nations contributing 8%. Advanced nations are expected to contribute 27% to the boost in worldwide GDP in the next 10 years, with the United States making the largest individual contribution at 12% [38].

The current article investigates the linkage between green trade, financial development, and shadow economies in EAGLE countries from 2003 to 2016. The authors conduct to answer the main questions using Bayesian method: *(i)* Does an advanced and inclusive financial system act as a deterrent to shadow economic activities, or does it inadvertently provide new channels for their proliferation? *(ii)* How does green trade affect informal activities? The study’s contributions will be outlined below. *Firstly*, there has been no prior investigation into the nexus between green trade and the shadow economy size. This is significant given the current trend of countries developing sustainable economies with environmentally beneficial commodity exchange activities. *Secondly*, there is limited empirical research on the connection between financial development and the activities of shadow economics. The examination of this linkage in the context of emerging countries is still an area of active interest. *Thirdly*, the article employs the Bayesian method, particularly advantageous when dealing with limited data. Bayesian methods provide a natural way to determine uncertainty in parameter estimates. Instead of producing point estimates, Bayesian models generate posterior distributions, which represent a range of plausible values for the parameters.

The rest of the current article is organized as follows: Section 2 describes the literature review on the linkage between green trade, financial development, and the magnitude of the shadow economy. Section 3 shows the dataset and methodology. Section 4 illustrates the empirical results. Section 5 concludes the findings and proposes policy implications.

## 2. Literature review

### 2.1. The relationship between green trade and the shadow economy

In the past few decades, there has been a global acknowledgment of the crucial need to protect environmental quality. The inclusion of environmental sustainability in the priorities of policymakers across numerous countries can be traced back to the initial United Nations Conference on the Human Environment, which took place in Stockholm in 1972 [39]. Numerous studies have shed light on the trend of cleaner production and consumption linked to sustainable economic growth [40, 41]. Subsequently, the focus on sustainability, particularly in harmonizing environmental and economic objectives, has taken a prominent role among major business entities. As a result, these firms are progressively embracing environmentally friendly practices.

In contrast to conventional trade, which encompasses the exchange of various goods and services without a specific focus on environmental factors, Charnovitz [42] initially introduced the term “green trade”. This concept refers to the trade of goods and services that adhere to specific environmental standards and criteria. While green trade has a substantial impact on informal economic activities, prior research has solely focused on examining the connection between trade and the shadow economy. In particular, employing a global dataset encompassing 116 nations from 2003 to 2014, Canh and Dinh Thanh [43] identified that the impacts of export multifariousness and the quality of export products exhibit non-linear patterns concerning the growth of the shadow economy. These nonlinear trends were consistently observed in low- and middle-income nations and high-income nations. In the long term, both enhanced export multifariousness and improved export quality could effectively diminish the scope of activities in the shadow economy. Meanwhile, Canh, Schinckus [12] employed two approaches, namely the panel-corrected standard errors estimator and the dynamic fixed effects autoregressive distributed lag (ARDL) estimator, to investigate the effects of trade liberalization on the shadow economy across a global dataset of 112 nations. During the period from 2005 to 2015, trade openness exerted a negative influence both in the short term and long term. Or Esaku [44] evaluated the connection between trade liberalization and participation in Uganda’s shadow economy using an ARDL bounds test technique. Their findings revealed that increased participation in international trade substantially diminished the magnitude of the shadow economy in Uganda. This suggests that as nations become more integrated into the global economy, businesses and individual businessmen are encouraged to operate within the official sector to take advantage of foreign market opportunities.

No previous studies have explored the nexus between green trade and the level of the shadow economy. Theoretically, based on Regulatory Compliance Theory, the growth and promotion of green trade can influence on reducing activities within the shadow economy. The core idea is that adherence to environmental regulations and standards in green trade creates a regulatory environment that makes it challenging for businesses involved in shadow economic activities to operate discreetly or avoid legal scrutiny [45, 46]. Therefore, businesses in the shadow economy may find it challenging to comply with these regulations, potentially encouraging a shift towards more legitimate, formal economic activities [47]. Besides, the nexus between green trade and the magnitude of the shadow economy is also elucidated by the principles of Consumer Behavior and Demand Theory. It posits that the growth of green trade, involving the exchange of environmentally sustainable goods and services, is influenced by consumer preferences for eco-friendly products. The theory suggests that as consumers become more environmentally conscious, there is a shift in demand towards products that align with sustainability principles [48, 49]. This shift, in turn, can impact businesses in the shadow economy as they respond to changing market dynamics. Moreover, the theory assumes that businesses responding to consumer demand for green products are incentivized to adopt environmentally sustainable practices [50, 51]. The green trade sector often encourages innovation and the adoption of new technologies to meet environmental standards. This emphasis on innovation may create economic opportunities for businesses to transition from the shadow economy into the formal sector by investing in environmentally friendly practices. Hence, businesses in the shadow economy may face challenges if they do not align with these green practices, as their products may become less desirable to environmentally conscious consumers.

On the contrary, from certain perspectives, green trade openness may indeed incentivize the shadow economy. First, green trade often comes with environmental standards and certifications that businesses need to adhere to [52–54]. The costs associated with meeting these standards, especially for small and informal businesses, can be significant [55]. In some cases, these businesses may choose to operate in the shadow economy to avoid the expenses and complexities of compliance [56, 57]. Some might find it easier to operate outside the formal system where they can avoid paperwork, inspections, and other regulatory burdens associated with environmentally friendly practices. Second, small businesses in developing economies might perceive green trade requirements as putting them at a competitive disadvantage compared to larger, more established enterprises. In response, some businesses may choose to operate informally to remain competitive. Third, there might be a gap between the demand for green products or services and the ability of informal businesses to meet those demands. If the formal market is dominated by larger, compliant businesses, informal or smaller entities might opt to operate in the shadow economy to cater to consumers who are less concerned about environmental standards. It’s crucial for policymakers to recognize these challenges and work towards creating an environment where businesses, especially smaller ones, can transition to green practices without facing insurmountable barriers. This involves addressing the costs of compliance, providing support for technology adoption, and raising awareness about the benefits of sustainability in the long term. Striking a balance between environmental goals and the economic realities of businesses is essential to minimize incentives for the shadow economy. From the above analysis, our hypothesis suggests that green trade has an adverse impact on the scope of shadow economy.

H_1_: Green trade has a negative impact on the size of the shadow economy.

### 2.2. The relationship between financial development and the shadow economy

Despite the rising significance of the shadow economy, there is a shortage of research exploring the influence of the progress of the financial sector on the extension of the shadow economy [58]. The financial sector plays a crucial function in consolidating savings, channeling resources toward the most productive investments, reducing information and transaction expenses, and fostering inter-industry trade. These dynamics result in enhanced efficiency in resource allocation, expedited technological advancements, and the accumulation of both material and human capital [59].

According to the Dual-Track Hypothesis, it shows a linkage between financial development and the magnitude of activities in the shadow economy, it is recommended that in nations with less developed financial systems, a dual-track economic structure may emerge. In such a structure, a formal track (regulated and monitored) and an informal track (shadow economy) coexist [60]. The official sector is characterized by financial institutions, regulatory frameworks, and established banking practices [61, 62]. The shadow sector, on the other hand, consists of economic activities that operate informally, often due to limited access to official financial services [63]. The dual-track structure may change as financial systems develop and become more inclusive. A rise in financial development, such as improvements in banking services, access to credit, and financial inclusivity, may lead to a diminution in the magnitude and significance of the shadow economy. Therefore, the hypothesis suggests that enhanced financial development provides alternatives for economic agents in the shadow economy to transition toward formal channels.

Countries burdened by elevated taxes and restrictive regulations create stronger incentives for engagement in informal economic activities. If the government effectively utilizes the financial sector for surveillance and tax transactions, financial development diminishes instances of evading taxation, thereby further alleviating the expansion of shadow economy activities [21, 64]. Additionally, enduring causes for informal development include institutions (Berdiev et al., 2018; Jahan et al., 2020). The financial sector represents a specific type of institution that is likely to influence the proliferation of the shadow economy [65]. More precisely, the financial sector plays several vital roles in an economy, including supplying entrepreneurs with access to essential credit and enabling the surveillance of business transactions for taxation goals. As a result, the development of the financial sector raises the opportunity cost associated with engaging in the shadow economy by reducing barriers to credit acquisition [66]. Consequently, it incentivizes informal entrepreneurs to shift toward legitimacy.

The empirical results about the effect of financial development on the level of activities in the shadow economy are a subject of ongoing controversy and debate. On the one hand, some studies suggest that the impact of financial sector development on informal activities is merely negative. For example, Canh and Thanh [67] investigated the impact of financial development on the magnitude of the shadow economy across various dimensions, including financial access, financial depth, and financial efficiency, and two sub-sectors, including financial market and financial institutions. Applying a balanced panel dataset covering 114 global economies from 2002 to 2015, key findings identified that nine financial indicators minimized the magnitude of the shadow economy. Notably, the study showed that the negative effects of financial development on the extension of the shadow economy were prominent in low and lower-middle-income nations and upper-middle-income nations, becoming dominant in high-income nations in the long run. Goudarzi and Mittone [68] revealed that individuals are inclined to engage in official economic activities to a greater extent when there is a higher level of financial development, even in cases where it is linked with elevated tax rates. Additionally, they observed that the composition of the shadow economy influences people’s inclination towards it, but this impact is only significant when financial development has reached an advanced stage, ensuring certainty in accessing credit.

On the other hand, the linkage between financial development and the extension of the shadow economy is observed from more complex angles. Imamoğlu, Katircioğlu [69] explored the repercussions of the development of the financial services sector on the scale of informal economic activities within European Union (EU) nations. The outcomes revealed a relationship characterized by an inverted U-shape between financial services and underground economic activities in the EU. In other words, at the early stages of financial development, unofficial economic activity exhibited a positive response, but as financial development progresses, this relationship turned negative within the EU. Consequently, this study concluded that the financial services sector significantly influences variations in the extent of informal economic activities in EU countries. Meanwhile, Gharleghi and Jahanshahi [70] used data from 29 developing and developed countries from 1975 to 2015 to show that a GDP per capita of US$33,600 was a good level of financial development. These indicators were liquid liabilities, private credit to deposit money banks, and stock market capitalization. The empirical findings revealed that above this threshold, financial development significantly contributed to diminishing the magnitude of the shadow economy. Conversely, for nations with per capita income below this threshold, financial development showed no impact. This suggested that nations with lower per capita incomes (below $33,600) should realize policies aimed at enhancing access to finance and credit markets. This improvement can lead to a substantial increase in per capita income, subsequently resulting in a reduction in the magnitude of activities in the shadow economy. Khan, Abdul Hamid [58] found that the relationship between financial development and the shadow economy was notably weaker in OIC nations compared to non-OIC nations. Therefore, our hypothesis posits that financial development exerts a detrimental influence on the activities of the shadow economy.

H_2_: Financial development has a negative impact on the size of the shadow economy.

## 3. Dataset and methodology

### 3.1. Models

In order to analyze the effect of financial development and green trade on the shadow economies of EAGLE countries, the empirical specification is constructed as follows:

$$SE_{i,t}=\alpha +\beta FD_{i,t}+\gamma GOP_{i,t}+\sum_{k=1}^{7}\varphi_{k}X_{i,t}+\varepsilon_{i,t}$$ (1)

where i represent the country; t indicates the year; SE denotes the activities of the shadow economy, estimated from the MIMIC or DGE models; FD denotes financial development; GOP denotes green trade; X represents other factors affecting the shadow economy, that is, the control variables; α, β, γ, and φ denote the regression coefficients, and ɛ is the stochastic disturbance term.

Seven main control variables were selected from the report of the previous research. (1) level of GDP growth (GDPgr) is represented by the annual percentage growth rate of GDP [71–73]. (2) As a measure of urbanization (URBA), we utilize the ratio of urban population to total population [74, 75]. (3) foreign direct investment (FDI) is measured by net inflows of FDI as a percentage of GDP [9, 10, 76]. (4) Government expenditure (EXPEND), measured as the general government’s final consumption expenditure as a percentage of GDP [73]. (5) KOF Globalization Index (KOFGI) so as to measure globalization [15, 17, 77]. (6) Social progress is measured by the Human Development Index (HDI) [3, 78]. (7) The level of corruption is measured by the Control of Corruption (CC) indicator [79–81]. The all variables of the current article have been summarized in Table 1.

**Table 1:** Variables’ description.

Variables	Symbol	Definitions	Sources
***Dependent variables***			
Shadow economy (SE)	MIMIC_SE	The magnitude of the shadow economy (% of official GDP) from Multiple indicators multiple causes model-based (MIMIC) estimates	Elgin et al. (2021)
Shadow economy (SE)	DGE_SE	The magnitude of the shadow economy (% of official GDP) from Dynamic general equilibrium model-based (DGE) estimates	Elgin et al. (2021)
***Independent variables***			
*Financial development*			
Financial development index	FD	Measures the access, magnitude, stability, and efficiency of the financial system. The FD lies between 0 and 1 (higher values denote higher financial development levels)	IMF
Financial institution development index	FI	Measures the access, magnitude, stability, and efficiency of financial institutions. The FI lies between 0 and 1 (higher values denote higher financial institution development levels)	IMF
Financial market development index	FM	Measures the access, magnitude, stability, and efficiency of financial markets. The FM lies between 0 and 1 (higher values denote higher financial market development levels)	IMF
*Green trade*			
Green Openness Index	GOP	GOP index is measured as the sum of a country’s green exports and imports as a share of that country’s GDP (in %)	IMF
***Control variables***			
GDP growth	GDPgr	GDP growth (annual %)	WDI
Urbanization	URBA	Urbanization level (urban population/total population)	WDI
Foreign direct investment	FDI	Foreign direct investment, net inflows as a percent of GDP	WDI
Government expenditure	EXPEND	General government final consumption expenditure as a percent of GDP	WDI
KOF Globalization Index	KOFGI	Measures globalization. The HDI lies between 1 and 100 with higher values indicating higher levels of globalization	KOF SEI
Social progress	HDI	Measures human development. The HDI lies between 0 and 1 (higher values denote higher human development levels)	UNDP
Control of Corruption	CC	Measures corruption on a score of between -2.5 and +2.5, where -2.5 = much corruption and 2.5 = less corruption	WGI

Note: IMF—International Monetary Fund; WDI—World Development Indicators; KOFSEI—KOF Swiss Economic Institute; UNDP—United Nations Development Programme; WGI—Worldwide Governance Indicators.

### 3.2. Dataset

Table 2 describes the descriptive statistics of all variables. The first variable reflects the average magnitude of the shadow economy of the selected EAGLE nations, which is 30.86% (MIMIC_SE) or 28.50% (DGE_SE), while the minimum is 11.40% (or 8.95%) and the maximum is 57.75% (or 54.12%). It is clearly seen that shadow economic activity in EAGLE countries accounts for 1/3 of official GDP on average. On the other hand, the financial development index (FD) is 0.39 on average, while the lowest index is 0.13 and the highest is 0.66. The average Green Openness Index (GOP) for EAGLE in the given period was 3.25, with a standard deviation of 2.62. Brief descriptions related to control variables are also displayed in Table 2.

**Table 2:** Descriptive statistics.

Variable	Obs	Mean	Std. dev.	Min	Max
MIMIC_SE	210	30.86	11.71	11.40	57.75
DGE_SE	210	28.50	10.80	8.95	54.12
FD	210	0.39	0.13	0.13	0.66
FI	210	0.37	0.14	0.16	0.70
FM	210	0.39	0.16	0.03	0.69
GOP	210	3.25	2.62	0.78	11.49
GDPgr	210	5.17	3.09	-7.80	14.23
URBA	210	53.46	18.56	25.43	86.04
FDI	210	2.35	1.63	-0.25	9.66
EXPEND	210	11.56	3.91	0.95	20.79
KOFGI	210	60.88	8.35	40.14	81.31
HDI	210	0.67	0.10	0.45	0.83
CC	210	-0.57	0.40	-1.50	0.41

### 3.3. Estimation method

Bayesian regression is a type of regression that employs Bayesian statistics to estimate the unknown parameters of a model. The goal of Bayesian regression is to ascertain the most optimal estimation of all parameters of a linear model that characterizes the link between the response and independent variables. Bayesian regression uses Bayes’ theorem to estimate the likelihood of a set of parameters given the observed dataset. The Bayes theorem [82] gives the linkage between an event’s prior probability and its posterior probability after evidence is taken into account as follows:

$$P(A|B)=\frac{P(B|A)xP(A)}{P(B)}$$ (2)

Here, A and B are to be events; P(A | B) is the likelihood that event A will happen, provided that event B has already transpired (Posterior); P(B | A) is the probability of event B happening given that event A has already transpired (Likelihood); P(A) is the probability of event A happening (Priority) and P(B) is the probability of event B happening. P(B) is the standardization constant; thus, Bayes’ law is present as a proportion:

P(A|B)=P(A|B)xP(A) (3)

According to Eq (3), the posterior distribution is the weighted mean of the knowledge of previous parameters for the dataset and the information about the parameters in the observed dataset.

The procedure for performing Bayesian regression is as follows: first, we employ a normal distribution prior because previous researchers consider that it provides a decent representation of the distribution of effects and the results of the Bayesian analysis of the study’s hypotheses will not be skewed either positively or negatively [83]. As a result, we employ the prior distribution with a mean of 0 and a variance of 1. Second, we suppose normal distributions prior of all parameters arrive from our econometric models. In the last step, we use simulations to change our previous beliefs. We use Markov Chain Monte Carlo (MCMC) methods and Gibbs sampling to make posterior distributions of all parameters. Because the posterior distribution provides a density function for all parameters, the convergence of MCMC is evaluated [84, 85].

## 4. Results and robustness checks

This section displays the basis as well as robustness check results of the influence of financial development and green international trade on the shadow economy in EAGLE nations. In the simulation, we execute two MCMC chains. For each MCMC chain, we extract 12,500 interactions from the posterior distribution and exclude the initial 2,500 interactions. The size of MCMC for each chain will be 10,000 and 20,000 for two chains.

### 4.1. Main findings

Table 3 reports the results of the impact of financial development and green trade on the magnitude of the shadow economy. Specifically, Column 3.1 examines the impact of financial development on the activities of the shadow economy, Column 3.2 examines the impact of green trade on the shadow economy, and Column 3.3 examines the simultaneous influence of financial development and green trade on the extension of activities in the shadow economy.

**Table 3:** The financial development, green international trade and shadow economy (dependent variable: MIMIC_SE).

Variable	(3.1)		(3.2)		(3.3)	
	Mean	Posterior probability	Mean	Posterior probability	Mean	Posterior probability
FD	-0.5690	0.7146	─	─	-0.3814	0.6501
GOP	─	─	-2.6238	1.0000	-2.6149	0.8938
GDPgr	-0.2514	0.8266	-0.2913	0.8984	-0.2896	0.9970
URBA	0.1048	0.9359	0.1637	0.9967	0.1646	0.5924
FDI	-1.2313	0.9959	-0.0962	0.5856	-0.0992	1.0000
EXPEND	-0.7361	0.9936	-1.4978	1.0000	-1.4895	1.0000
KOFGI	0.6087	1.0000	0.8118	1.0000	0.8110	0.5728
_cons	0.6888	0.7561	0.1733	0.5703	0.1825	
Variance	136.9851	─	99.1928	─	99.3165	─
**MCMC diagnostics**						
ESS min	19,243		18,629		18,212	
Rc max	1.0002		1.0001		1.0002	

Note: Markov Chain Monte Carlo (MCMC); effective sample size (ESS); Gelman and Rubin R-statistic (Rc).

The results for the financial development variable (FD) are in line with our expectations. The probability that the variable FD has a negative effect is 71.46% (column 3.1) or 65.01% (column 3.3). The mean coefficients are β = -0.5690 or β = -0.3814. In sum, we detect strong evidence for the negative effect of financial development on the scope of the shadow economy. This indicates that raising financial sector development will reduce the magnitude of the shadow economy in EAGLE countries.

As can be seen in Table 3, we obtain a negative and strong association between the shadow economy and green trade variable (GOP). The probability that the variable GOP has a negative effect is 100% (column 3.2) or 89.38% (column 3.3). The mean coefficients are γ = -2.6238 or γ = -2.6149. This implies that EAGLE nations with more green trade activities are more likely to have a smaller shadow economy.

In the case of control variables, we find that GDPgr, FDI, and EXPEND have a strong and negative influence on the magnitude of activities in the shadow economy, whereas the variables URBA, and KOFGI seem to exert a strong positive effect on the growth of activities in the shadow economy. Earlier research identified associations between the shadow economy and similar variables, such as Ajide and Dada [15], Tran, Tran [73], Huynh and Nguyen [86], Ajide and Adeniyi [87] and Kireenko and Nevzorova [88].

Besides, the results of MCMC convergence diagnostics are also presented in this table. First, the results show that the minimum effective sample size (ESS) ranges from 18,212 to 19,243, which is approximately approaching 20,000. Second, the highest Rc value is 1.0002. Thus, the MCMC diagnostic results suggest that the algorithm is convergent (Gelman & Rubin, 1992; Kruschke, 2010).

Next, we estimate the impact of two components of financial development, including financial institution development (FI) and financial market development (FM), on the shadow economy, as delineated in Table 4. In column 4.1, we show a delineation of the effect of FI on the shadow economy. In column 4.2, we show a report on the effect of FM on the shadow economy. In column 4.3, we present a report on the effect of the FI, FM, and GOP on the shadow economy.

**Table 4:** The two sub-indices of financial development, green international trade and shadow economy (dependent variable: MIMIC_SE).

Variable	(4.1)		(4.2)		(4.3)	
	Mean	Posterior probability	Mean	Posterior probability	Mean	Posterior probability
FI	-0.6023	0.7288	─	─	-0.3423	0.6353
FM	─	─	-0.5192	0.7020	-0.3943	0.6547
GOP	─	─	─	─	-2.6114	1.0000
GDPgr	-0.2544	0.8349	-0.2512	0.8283	-0.2916	0.8991
URBA	0.1063	0.9381	0.1035	0.9334	0.1642	0.9968
FDI	-1.2467	0.9966	-1.2404	0.9955	-0.0899	0.5833
EXPEND	-0.7389	0.9938	-0.7393	0.9937	-1.4844	1.0000
KOFGI	0.6086	1.0000	0.6106	1.0000	0.8123	1.0000
_cons	0.6832	0.7559	0.6746	0.7531	0.1782	0.5705
Variance	136.8256	─	137.0093	─	98.9897	─
**MCMC diagnostics**						
ESS min	18,411		18,434		18,256	
Rc max	1.0002		1.0004		1.0002	

Note: Markov Chain Monte Carlo (MCMC); effective sample size (ESS); Gelman and Rubin R-statistic (Rc).

The mean coefficient of variable FI is -0.6023 (column 4.1) or -0.3423 (column 4.3), with the probability that the variable FI has a negative effect ranging from 63.53% to 72.88%. The results display that financial institutions development have a negative and significant effect on the level of the shadow economy. The mean coefficient of variable FM is -0.5192 (column 4.2) or -0.3943 (column 4.3), with the probability that the variable FM has a negative effect ranging from 65.47% to 70.20%. This indicates that the higher the degree of financial market development, the smaller the shadow economy is. These findings conform with conclusions of Canh and Thanh [67]. The influence of the shadow economy was more profoundly influenced by financial institutions, particularly their efficiency, than by financial markets. However, they argued that the shadow economy experienced a positive influence from both financial depth and financial access in the short term, whereas financial institutions seemed to decrease the shadow economy in the long run. Table 4 also displays that the GOP has a strong negative relationship with the shadow economy.

### 4.2. Robustness checks

The robustness of the basic analysis was checked by using a substitute measure of the extension of the shadow economy and additional control variables. The results can be found in Tables 5–7.

**Table 5:** Robust checks: An alternative measure of the scope of the shadow economy (dependent variable: DGE_SE).

Variable	(5.1)		(5.2)		(5.3)	
	Mean	Posterior probability	Mean	Posterior probability	Mean	Posterior probability
FD	-0.5976	0.7218	─	─	-0.4001	0.6567
GOP	─	─	-2.3868	1.0000	-2.3898	1.0000
GDPgr	-0.1508	0.7281	0.2176	0.8074	-0.1860	0.8057
URBA	0.1013	0.9403	0.0560	0.9972	0.1567	0.9969
FDI	-1.1081	0.9936	0.4168	0.5366	-0.0339	0.5286
EXPEND	-0.6991	0.9938	0.2569	1.0000	-1.3964	1.0000
KOFGI	0.5521	1.0000	0.0619	1.0000	0.7374	1.0000
_cons	0.7564	0.7757	0.9937	0.5917	0.2436	0.5949
Variance	119.3072	─	8.9057	─	88.5321	─
**MCMC diagnostics**						
ESS min	18,329		18,600		19,036	
Rc max	1.0002		1.0001		1.0003	

Note: Markov Chain Monte Carlo (MCMC); effective sample size (ESS); Gelman and Rubin R-statistic (Rc).

**Table 6:** Robust checks: Add social progress and control of corruption (dependent variable: MIMIC_SE).

Variable	(6.1)		(6.2)		(6.3)	
	Mean	Posterior probability	Mean	Posterior probability	Mean	Posterior probability
FD	-0.5329	0.7022	─	─	-0.3507	0.6348
GOP	─	─	-2.4194	1.0000	-2.4146	1.0000
GDPgr	-0.2781	0.8661	-0.3036	0.9122	-0.3051	0.9149
URBA	0.1296	0.9748	0.1755	0.9984	0.1754	0.9985
FDI	-1.1391	0.9946	-0.1124	0.6011	-0.1029	0.5958
EXPEND	-0.6992	0.9938	-1.4177	1.0000	-1.4091	1.0000
KOFGI	0.5559	1.0000	0.7665	1.0000	0.7669	1.0000
HDI	-0.4824	0.6897	-0.6056	0.7282	-0.6155	0.7316
CC	-3.3829	1.0000	-2.0479	0.9886	-2.0443	0.9896
_cons	0.5682	0.7149	0.0929	0.5370	0.0870	0.5315
Variance	121.6432	─	95.2319	─	95.1536	─
**MCMC diagnostics**						
ESS min	16,376		17,670		17,765	
Rc max	1.0008		1.0002		1.0002	

Note: Markov Chain Monte Carlo (MCMC); effective sample size (ESS); Gelman and Rubin R-statistic (Rc).

**Table 7:** Robust checks: Add social progress and control of corruption (dependent variable: DGE_SE).

Variable	(7.1)		(7.2)		(7.3)	
	Mean	Posterior probability	Mean	Posterior probability	Mean	Posterior probability
FD	-0.5413	0.7087	─	─	-0.3369	0.6347
GOP	─	─	-2.1716	1.0000	-2.1675	1.0000
GDPgr	-0.1790	0.7808	-0.2003	0.8300	-0.1997	0.8280
URBA	0.1271	0.9805	0.1689	0.9991	0.1687	0.9991
FDI	-0.9968	0.9910	-0.0572	0.5539	-0.0518	0.5557
EXPEND	-0.6601	0.9921	-1.3190	1.0000	-1.3092	1.0000
KOFGI	0.4966	1.0000	0.6888	1.0000	0.6887	1.0000
HDI	-0.5098	0.6939	-0.6404	0.7431	-0.6448	0.7424
CC	-3.5530	1.0000	-2.1963	0.9944	-2.1874	0.9923
_cons	0.6120	0.7342	0.1322	0.5539	0.1362	0.5569
Variance	104.3769	─	84.4417	─	84.3268	─
**MCMC diagnostics**						
ESS min	15,931		17,461		17,545	
Rc max	1.0002		1.0002		1.0001	

Note: Markov Chain Monte Carlo (MCMC); effective sample size (ESS); Gelman and Rubin R-statistic (Rc).

#### 4.2.1. Another measure of the shadow economy

First, we utilize a substitute measure of the level of the shadow economy estimated using DGE. Table 5 reports the estimates of the Bayesian regression with DGE_SE as the dependent variable. The results regarding the impact of financial development and green international trade are align with the previous results. Both financial development and green trade have a negative and strong effect on the shadow economy, meaning that a more developed financial economy and green products in international trade are associated with a lower shadow economy.

#### 4.2.2. Include supplementary control variables

Moreover, to diminish the bias of missing variables, in Tables 6 and 7, we control for other effects to support the primary findings. Noticeably, we insert two factors, including social progress and corruption. We give some reasons for the selection of the additional two control variables. Medda, Palmisano [89] provide a hypothesis to explain the linkage between social progress and the activities of the shadow economy, and their results indicate that social progress, measured through the HDI, has a strong negative impact on the shadow economy. Esaku [81], Goel and Saunoris [90] showed that the higher the level of control over corruption, the lower the scope of the shadow economy.

The results delineated in Tables 6 and 7 are qualitatively the same as those reported in Tables 3 and 5. Particularly, all the mean coefficients associated with financial development (FD) and green international trade (GOP) variables are negative, and the probability that the variables FD and GOP have a negative effect is above 60%, meaning that financial development and green international trade activities reduce the magnitude of the shadow economy in EAGLE nations. Thus, our results are robust to the inclusion of two control variables.

### 4.3. Discussion

First, the first empirical results show that promoting the growth of the financial sector will diminish the scale of the shadow economy in EAGLE nations, in keeping with the findings of Blackburn, Bose [21], Goudarzi and Mittone [68], Gharleghi and Jahanshahi [70], Berdiev and Saunoris [91]. A well-developed financial sector provides better access to formal banking and financial services in EAGLE countries [92, 93]. Financial sector development typically involves the availability of credit and financing options for businesses. When legitimate financing channels are accessible, businesses may be less inclined to rely on informal sources of funding that are often associated with the shadow economy [94]. Therefore, when individuals and businesses have easier access to banking, they are more likely to engage in official economic activities, reducing the need to operate in the shadow economy. Moreover, the advanced financial sector can facilitate more effective tax collection mechanisms in this context. With improved tax systems and enforcement, individuals and businesses are more likely to comply with tax obligations, reducing the scope of the underground economy where transactions often go unreported. Besides, the growth of the financial sector often involves the adoption of digital technologies [95–97]. Digital payment systems, online banking, and electronic financial transactions provide traceable records. This digital trail makes it more challenging to engage in off-the-books activities characteristic of the informal economy [98]. The extension of the financial sector is also often associated with broader economic development, leading to job creation and increased income opportunities. As legitimate employment options expand, individuals are less likely to engage in informal economic activities to sustain their livelihoods.

Second, the authors explore that involvement in increased green trade is likely to result in a diminished shadow economy. EAGLE countries, recognizing the importance of sustainable development, often establish strict regulatory frameworks for green industries. These regulations create a more transparent business environment, as companies engaged in green trade are required to comply with environmental standards, report their activities, and adhere to legal and tax obligations [99]. This increased scrutiny reduces the likelihood of engaging in shadow economic activities. Moreover, many EAGLE countries are signatories to international agreements and conventions related to environmental sustainability [100]. Engaging in green trade allows these countries to fulfill their global commitments. Maintaining a positive international reputation becomes crucial, and adherence to ethical business practices and transparency helps build trust with international partners, investors, and organizations. Therefore, this discourages organizations and enterprises from engaging in the shadow economy. Furthermore, governments in EAGLE nations often provide incentives and support for businesses engaged in green trade as part of their commitment to sustainable development. To access these incentives, companies must comply with regulations and operate transparently. This further discourages engagement in the shadow economy to evade formal requirements.

## 5. Conclusion and policy implications

This article reaches the primary conclusions about the linkage between green trade, financial development, and the level of activities in the shadow economy by employing the Bayesian regression model within EAGLE countries from 2003 to 2016: *(i)* The advancement of the financial sector will diminish the magnitude of the shadow economy. Particularly, the growth of financial institutions and financial markets exerts a noteworthy and adverse influence on the scope of activities in the shadow economy; *(ii)* Participating more in green trade is likely to lead to a reduced shadow economy; *(iii)* Economic indicators such as GDP growth, FDI, and government expenditure negatively influence the magnitude of the shadow economy, while urbanization and globalization exhibit a robust positive impact on the shadow economy.

In light of the empirical findings, specific policy implications are proposed, as outlined:

Firstly, governments should focus on strengthening financial institutions, including banks and non-banking financial entities, to enhance their capacity to provide formal financial services. A well-functioning financial sector provides individuals and businesses with access to credit, savings, and other financial services, reducing the need for informal financial channels. It is vital to implement policies that facilitate access to formal credit for individuals and businesses, particularly those in the informal sector. Improved access to formal credit can help entrepreneurs and small businesses transition from informal to formal economic activities by providing them with the necessary capital to invest and expand their operations. Besides, EAGLE countries should continuously assess and reform regulatory frameworks to create an environment conducive to formal economic activities. Regulatory burdens and complexities can drive businesses and individuals towards the informal sector. Streamlining regulations, reducing bureaucratic hurdles, and enhancing the ease of doing business can incentivize formalization. Implement and enforce anti-corruption measures to improve governance and reduce the prevalence of informal payments are essential because corruption often drives economic activities underground. By combating corruption, governments can create a more transparent and accountable business environment, encouraging formalization. Additionally, high and complex tax structures can incentivize businesses and individuals to operate in the shadow economy. Streamlined and fair tax policies can reduce these incentives. Therefore, EAGLE nations need to review and reform tax policies to make them fair, simple, and supportive of formal economic activities. In summary, policies that support financial development and formalization in EAGLE countries should be comprehensive, addressing both economic and regulatory aspects while considering the unique characteristics of each country within the EAGLE group.

Secondly, these countries face various challenges, including issues related to the shadow economy and the need for sustainable development. To enhance green trade and reduce the magnitude of activities in the shadow economy in the context of EAGLE nations, policymakers could consider implement and strengthen policies that promote green trade, including incentives for sustainable practices, renewable energy adoption, and environmentally friendly production processes. The governments can strengthen and enforce environmental regulations to ensure that businesses operate in an eco-friendly manner. In particular, EAGLE countries should develop monitoring systems to track and penalize companies engaging in environmentally harmful practices, while also facilitating trade agreements that prioritize and reward adherence to environmental standards.

## Supporting information

S1 Table List of current EAGLE countries. (PDF)

S2 Table The size of the shadow economy in EAGLE countries. (PDF)
