# Supplementary material for: Impacts of financial development and green trade on the shadow economy: An insight of eagle countries using Bayesian approaches
Source: PLoS One. 2024 May 28;19(5):e0303135. doi: 10.1371/journal.pone.0303135 (PMC11132503; doi:10.1371/journal.pone.0303135)
Supplement: S1 Table — (PDF) [file pone.0303135.s001.pdf]

S1 Table: List of current EAGLE countries

No.	EAGLEs members
1	Bangladesh
2	Brazil
3	People's Republic of China
4	Egypt
5	India
6	Indonesia
7	Iran
8	Malaysia
9	Mexico
10	Nigeria
11	Pakistan
12	Philippines
13	Russia
14	Turkey
15	Vietnam
