# Supplementary material for: Impacts of financial development and green trade on the shadow economy: An insight of eagle countries using Bayesian approaches
Source: PLoS One. 2024 May 28;19(5):e0303135. doi: 10.1371/journal.pone.0303135 (PMC11132503; doi:10.1371/journal.pone.0303135)
Supplement: S2 Table — (PDF) [file pone.0303135.s002.pdf]

S2 Table: The size of the shadow economy in EAGLE countries

MIMIC_SE (percent of GDP: mean = 30.86)			
Highest		***Lowest***	
Nigeria	56.26	China	11.91
Russia	43.90	Vietnam	14.75
Philippines	40.06	Iran	17.63
**DGE_SE (percent of GDP: mean = 28.50)**			
Highest		***Lowest***	
Nigeria	51.61	China	11.04
Russia	40.37	Vietnam	13.84
Philippines	37.67	Iran	16.80
